# Retinal ganglion cell survival and axon regeneration in *Wld*^*S*^ transgenic rats after optic nerve crush and lens injury

**DOI:** 10.1186/1471-2202-13-56

**Published:** 2012-06-06

**Authors:** Barbara Lorber, Alessia Tassoni, Natalie D Bull, Keith R Martin

**Affiliations:** 1 Centre for Brain Repair, University of Cambridge, Cambridge, UK; 2 First Department of Ophthalmology, Evgenidion Hospital, University of Athens, Athens, Greece; 3 Cambridge NIHR Biomedical Research Centre, Cambridge, UK; 4 Eye Department, Addenbrooke's Hospital, Cambridge, UK; 5 Cambridge Centre for Brain Repair, University of Cambridge, Cambridge, CB2 0PY, United Kingdom

**Keywords:** Slow Wallerian degeneration mutation, Retinal ganglion cell, Axon regeneration, Survival, Activated retinal glia

## Abstract

**Background:**

We have previously shown that the slow Wallerian degeneration mutation, whilst delaying axonal degeneration after optic nerve crush, does not protect retinal ganglion cell (RGC) bodies in adult rats. To test the effects of a combination approach protecting both axons and cell bodies we performed combined optic nerve crush and lens injury, which results in both enhanced RGC survival as well as axon regeneration past the lesion site in wildtype animals.

**Results:**

As previously reported we found that the *Wld*^*S*^ mutation does not protect RGC bodies after optic nerve crush alone. Surprisingly, we found that *Wld*^*S*^ transgenic rats did not exhibit the enhanced RGC survival response after combined optic nerve crush and lens injury that was observed in wildtype rats. RGC axon regeneration past the optic nerve lesion site was, however, similar in *Wld*^*S*^ and wildtypes. Furthermore, activation of retinal glia, previously shown to be associated with enhanced RGC survival and axon regeneration after optic nerve crush and lens injury, was unaffected in *Wld*^*S*^ transgenic rats.

**Conclusions:**

RGC axon regeneration is similar between *Wld*^*S*^ transgenic and wildtype rats, but *Wld*^*S*^ transgenic rats do not exhibit enhanced RGC survival after combined optic nerve crush and lens injury suggesting that the neuroprotective effects of lens injury on RGC survival may be limited by the *Wld*^*S*^ protein.

## Background

The visual system provides an excellent model for studying neuronal survival and axon regeneration after central nervous system injury. Following optic nerve lesion, axotomised adult rat retinal ganglion cells (RGC) die in large numbers. The surviving RGC only show a transient sprouting reaction with very limited axon growth past the lesion site [1]. However, it has been found that RGC survival and axon regeneration past the optic nerve lesion site can be enhanced, for example by lens injury [2-4], peripheral nerve injury [1,4], or intravitreal zymosan injection [2]. This is correlated with invasion of macrophages into the eye and activation of retinal glia, astrocytes and Müller glia [2,4,5], which produce a variety of growth factors, including oncomodulin [6,7] and ciliary neurotrophic factor/apolipoprotein E respectively [5,8]. The role of macrophages in mediating RGC survival and axon regeneration has recently been challenged [9] and activated retinal glia have been shown to be important mediators of this response [5,8].

We have previously shown that the slow Wallerian degeneration mutation (*Wld*^*S*^) delays RGC axon degeneration in adult rats after optic nerve injury, but does not reduce cell body death compared to wildtype animals [10]. To test the effects of a combination approach protecting both axons and cell bodies we performed combined optic nerve crush and lens injury, which enhances RGC survival as well as axon regeneration in wildtype rats [2-4]. This is also of particular interest in terms of the effects of the *Wld*^*S*^ mutation on the axon regeneration potential in the central nervous system (CNS) because previous studies have mainly focused on peripheral nervous system (PNS) regeneration in mice, in which the mutation first arose [11,12]. Whilst there have been reports that axonal regeneration is impaired in *Wld*^*S*^ transgenic mice, it has also been shown that they are able to regenerate normally in an appropriate environment [11,13,14].

Interestingly, we found that whilst optic nerve crush and lens injury did not lead to enhanced RGC survival in *Wld*^*S*^ transgenic rats, these cells were nevertheless able to successfully re-grow RGC axons past the optic nerve lesion site.

## Results

We have previously confirmed that transgenic *Wld*^*S*^ rat RGC express the *Wld*^*S*^ protein, and that their axons show delayed Wallerian degeneration after optic nerve crush [10].

### RGC axon regeneration

**Figure 1:**
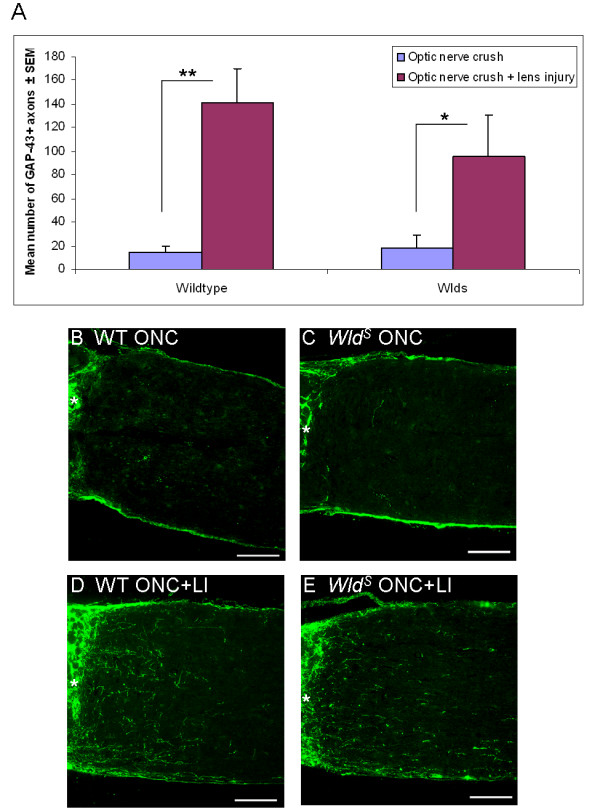
**RGC axon regeneration is similar between *Wld ***^***S ***^**transgenic and wildtype rats after optic nerve crush and lens injury.** (**A**) Quantification of the mean number of RGC axons extending 250 μm past the optic nerve lesion site 14 days after either optic nerve crush alone, or in combination with lens injury in adult wildtype or *Wld*^*S*^ transgenic rats. Significant differences are indicated by asterisk (P < 0.05 = *; P < 0.01 = **). Images of RGC axon regeneration past the optic nerve lesion site (indicated by asterisk), 14 days after (**B, C**) optic nerve crush alone or (**D, E**) in combination with lens injury in wildtype and *Wld*^*S*^ transgenic rats. WT = Wildtype; ONC = Optic nerve crush; ONC + LI = Optic nerve crush + lens injury; Scale bars: 100 μm

Two weeks after optic nerve crush alone, only a small number of adult rat RGC axons were able to grow past the optic nerve lesion site in both wildtype and *Wld*^*S*^ transgenic rats with no significant difference between the two (Figure 1A-C). However, optic nerve crush combined with lens injury significantly increased the number of RGC axons crossing the optic nerve lesion site and growing into the distal optic nerve stump at 14 days post-injury. This regeneration response, whilst appearing slightly stronger in wildtype rats (wildtype: P < 0.01, *Wld*^*S*^: P < 0.05 versus optic nerve crush), was not significantly different to that found in *Wld*^*S*^ transgenic rats (Figure 1A,D,E). Therefore, RGC axon regeneration was similar between wildtype and *Wld*^*S*^ transgenic rats.

### RGC survival

For ease of RGC identification we used Islet-1, which is expressed by RGC as well as by displaced amacrine cells in the ganglion cell layer. Islet-1 is also expressed by some cells in the inner nuclear layer [15,16]. However, as it was previously shown that amacrine cells are not affected by optic nerve lesions [17,18], changes in Islet-1 labelling in the ganglion cell layer are likely to be due to changes in the number of surviving RGC, though we can not exclude that lens injury may have had an effect on amacrine survival.

**Figure 2:**
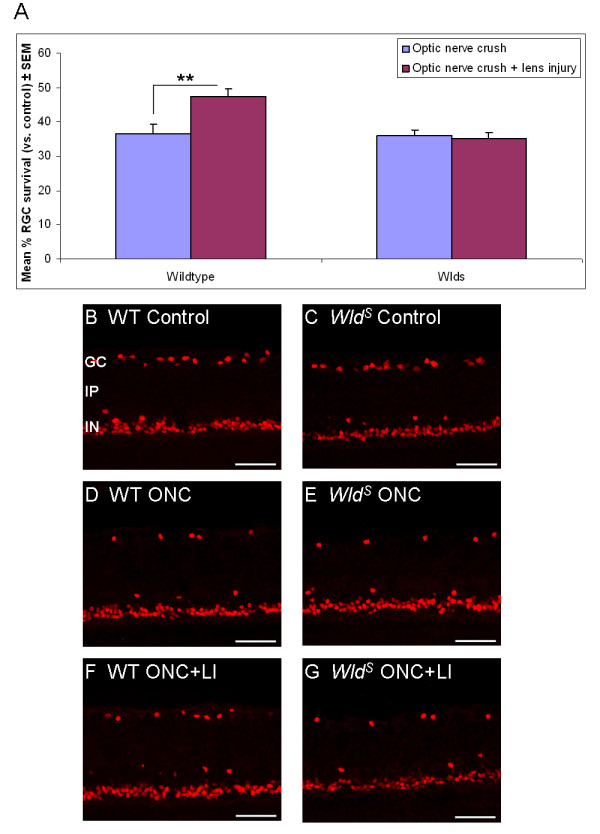
**Enhanced RGC survival does not occur in *Wld ***^***S ***^**transgenic rats after optic nerve crush and lens injury.** (**A**) Percentage of surviving RGC (compared to control) 14 days after either optic nerve crush alone or in combination with lens injury in adult wildtype and *Wld*^*S*^ transgenic rats. Significant differences are indicated by asterisk (P < 0.01 = **). Cross-sections through adult wildtype or *Wld*^*S*^ retinae stained for Islet-1 (to identify RGC in the ganglion cell layer) from (**B, C**) control (untreated), or 14 days after (**D, E**) optic nerve crush, or (**F, G**) optic nerve crush in combination with lens injury. GC = Ganglion cell layer; IP = Inner plexiform layer; IN = Inner nuclear layer; WT = Wildtype; ONC = Optic nerve crush; ONC + LI = Optic nerve crush + lens injury; Scale bars: 50 μm

We found that, as previously reported [10], two weeks after optic nerve crush, RGC survival was reduced to a third of control retinas and did not differ between wildtype and *Wld*^*S*^ transgenic rats. Interestingly, whilst lens injury at the time of optic nerve crush led to a significant increase in RGC survival (P < 0.01) in wildtype rats, it did not affect RGC survival in *Wld*^*S*^ transgenic rats (Figure 2A-G). Thus, whilst RGC survival was similar in both genotypes following optic nerve crush alone, optic nerve crush combined with lens injury revealed that, in contrast to wildtypes, no enhanced RGC survival response occurred in *Wld*^*S*^ transgenic rats.

### Retinal glia activation

**Figure 3:**
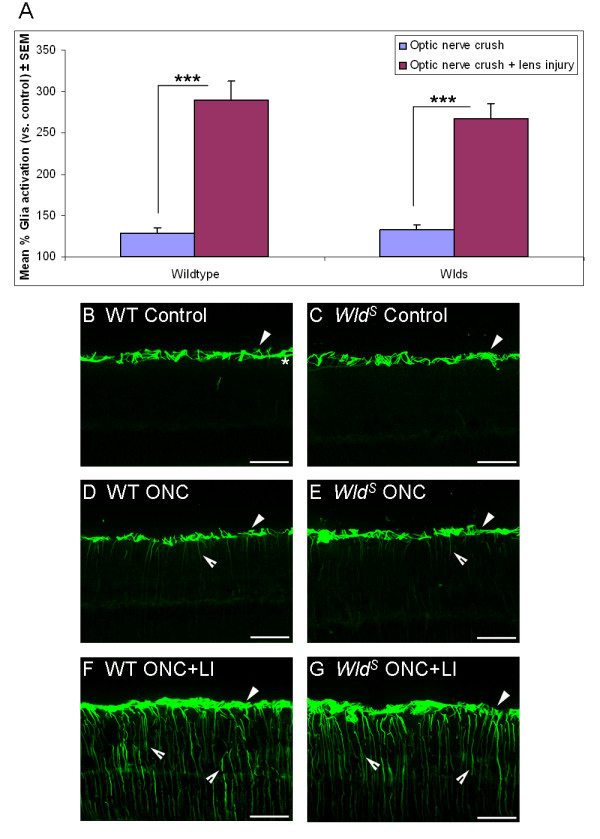
**Retinal glia activation is similar between *Wld ***^***S ***^**transgenic and wildtype rats after optic nerve crush and lens injury.** (**A**) Retinal glia activation (measured as percentage increase in GFAP intensity compared to control; control set as 100% baseline) 14 days after either optic nerve crush alone or in combination with lens injury in adult wildtype and *Wld*^*S*^ transgenic rats. Significant differences are indicated by asterisk (P < 0.001 = ***). Cross-sections through adult wildtype or *Wld*^*S*^ retinae stained for presence of GFAP + astrocytes (arrowheads)/Müller glia (open arrowheads) from (**B, C**) control (untreated), or 14 days after (**D, E**) optic nerve crush, or (**F, G**) optic nerve crush in combination with lens injury. The RGC layer is marked in panel B by an asterisk. WT = Wildtype; ONC = Optic nerve crush; ONC + LI = Optic nerve crush + lens injury; Scale bars: 50 μm

In both untreated wildtype and *Wld*^*S*^ transgenic rats expression of glial fibrillary acidic protein (GFAP) was restricted to astrocytic processes in the nerve fibre layer of the retina (Figure 3B,C). In both genotypes, fourteen days after optic nerve crush alone occasional Müller glia had become activated as evidenced by increased GFAP expression, with cell processes crossing the retinal layers (Figure 3A,D,E). Combination of optic nerve crush with lens injury led to a significant increase in retinal glia activation over that observed in optic nerve crush alone, in both wildtype (P < 0.001) and *Wld*^*S*^ transgenic rats (P < 0.001) with pronounced GFAP expression visible in Müller glia throughout the retina (Figure 3A,F,G). Retinal glia activation after optic nerve crush was not significantly different between *Wld*^*S*^ transgenic and wildtype rats. Thus, activation of retinal glia occurred to a similar extent in wildtype and *Wld*^*S*^ transgenic rats.

## Discussion

The *Wld*^*S*^ mutation has long been linked with delayed Wallerian degeneration in the PNS and CNS after injury [10-12] but relatively few studies have looked at the axon regenerative potential in *Wld*^*S*^ transgenic animals. Almost all of these studies have focused on *Wld*^*S*^ transgenic mice and investigated the regeneration potential after peripheral nerve injury [11,13,14], with only one study looking at recovery after injury to the CNS. Whilst this study found that locomotor recovery after partial spinal cord injury was delayed in *Wld*^*S*^ transgenic mice, it was suggested that the speed with which recovery occurred in wildtype mice was likely to result from changes in functional plasticity rather than axon regeneration [19].

The present study is therefore the first to look at axon regeneration of *Wld*^*S*^ transgenic rats, a recently generated strain [20], after injury to the CNS using a unique model, the optic nerve crush model, in which it is possible to stimulate successful RGC axon regeneration in wildtype rats when optic nerve crush is combined with lens injury [2-4]. Interestingly, we found that whilst the regeneration response was slightly stronger in wildtype rats, *Wld*^*S*^ transgenic rats were able to successfully grow RGC axons past the optic nerve lesion site after optic nerve crush and lens injury, with regeneration not being significantly different from wildtypes. We also found that activation of retinal glia, previously shown to mediate RGC survival and axon regeneration response after optic nerve crush and lens injury in wildtype rats [5,8], occurred to a similar extent between *Wld*^*S*^ transgenic and wildtype rats. This finding shows that successful CNS axon regeneration can indeed occur in *Wld*^*S*^ transgenic rats.

Delayed Wallerian degeneration in the CNS compared to the PNS has long been associated with axon regenerative failure after CNS injury [21]. We have recently shown that in *Wld*^*S*^ transgenic rats Wallerian degeneration after optic nerve injury is also delayed [10]. Successful RGC axon regeneration through the normally inhibitory environment of the distal optic nerve in wildtype rats after lens injury has been associated with an altered growth-disinhibitory phenotype, potentially through release of metalloproteinases from regenerating RGC axons, which degrade myelin-derived inhibitory ligands in the distal optic nerve stump [22]. This may also explain why successful RGC axon regeneration induced by lens injury can occur in *Wld*^*S*^ transgenic rats where Wallerian degeneration is even further delayed.

Our findings are in line with a study in *Wld*^*S*^ transgenic mice where the *Wld*^*S*^ mutation did not appear to alter the growth phenotype compared to wildtype mice, as sensory dorsal root ganglion axons regenerated normally, although only in an optimal environment where Wallerian degeneration was experimentally enhanced [14].

Whilst RGC axon regeneration past the optic nerve lesion site was similar between *Wld*^*S*^ transgenic rats and wildtypes, we found that the significantly enhanced RGC survival that occurred in wildtype rats after optic nerve crush and lens injury did not occur in *Wld*^*S*^ transgenic rats. These results could be consistent with *Wld*^*S*^ transgenic rats indeed having an enhanced RGC axon regeneration potential compared to wildtype rats, but this will need to be investigated more fully in future studies. It may seem surprising that, despite an absence of enhanced RGC survival in *Wld*^*S*^ transgenic rats after optic nerve crush and lens injury, successful RGC axon regeneration past the lesion site occurred. However, this finding is consistent with other studies which show that survival and regeneration are not linked *per se*, and that these two responses to injury appear to be regulated independently and can be modulated by distinct intracellular pathways [23,24].

As previously reported [10], RGC survival two weeks after optic nerve crush alone was similarly reduced to a third of control eyes in both wildtype and *Wld*^*S*^ transgenic rats, suggesting that there is no somatic RGC protection conferred through *Wld*^*S*^ in this model. This is in line with another study [25] which showed that there was no direct protective effect on cell bodies after induction of cell death in L4 motoneurons in *Wld*^*S*^ transgenic rats, suggesting that the observed neuroprotective effect in other injury models [12,26] was most likely due to secondary axonal protection. Whilst we did not observe a neuroprotective effect of the *Wld*^*S*^ protein on RGC survival in this and a previous study [10] after optic nerve crush, it is possible that secondary axonal protection may have made RGC from *Wld*^*S*^ transgenic rats less responsive to survival factors released in response to lens injury.

Several mechanisms have been suggested to mediate neuroprotection in *Wld*^*S*^ transgenic animals and it will be interesting to investigate in future if they interfere with RGC survival pathways activated through lens injury, thereby rendering the latter inactive in *Wld*^*S*^ transgenic rats. *Wld*^*S*^ is a fusion protein that consists of nicotinamide mononucleotide adenylyl-transferase-1 (Nmnat1) and a short fragment of the ubiquitin assembly protein UFD2. Whilst several reports have suggested that Nmnat is the key mediator of the neuroprotective *Wld*^*S*^ effects through upregulation of nicotinamide adenine dinucleotide levels, there are some reports that have challenged this view reviewed in [27,28]. Other proposed mechanisms through which *Wld*^*S*^ may confer neuroprotection include changes in gene expression, such as downregulation of the pituitary tumor transforming gene [29], or changes in the regulation of the cell cycle status which may “prime” neurons against future neurodegenerative insults by modifying endogenous stress pathways [30]. The latter findings were observed in uninjured *Wld*^*S*^ mouse cerebella so it will be interesting to examine to what extent changes in cell cycle status occur in *Wld*^*S*^ transgenic RGC and to study the effects that optic nerve crush and lens injury has on them.

There is, therefore, an array of mechanisms that have been suggested to underlie *Wld*^*S*^ mediated neuroprotection, with most studies however conducted in *Wld*^*S*^ transgenic mice [27]. It will be important to explore in future studies whether the same mechanisms are present in *Wld*^*S*^ transgenic rats. In that respect it will also be important to investigate further the role that non-neuronal retinal cells play, in particular glial cells which are mediators of the neuroprotective responses after lens injury [5,8]. The majority of proteins implicated in mediating mechanisms underlying neuroprotection in *Wld*^*S*^ transgenic animals are located in transgenic *Wld*^*S*^ neurons and their axons, with only some weak expression observed in glia [29,31]. In contrast to *Wld*^*S*^ transgenic mice, we previously found that retinal *Wld*^*S*^ expression also localizes to cells of the inner nuclear rat retina [10], an area where Müller glia cell bodies are also located. It will therefore be important to determine whether the expression of *Wld*^*S*^ and its downstream effector proteins indeed correlate to Müller glia, and whether optic nerve crush combined with lens injury modulates their expression. Such future investigations will further our understanding of the mechanisms that underlie the RGC survival and axon regeneration responses in the *Wld*^*S*^ transgenic rat model.

## Conclusions

In summary we have shown that whilst RGC axon regeneration is similar between *Wld*^*S*^ transgenic and wildtype rats, somatic RGC protection is not conferred through, and may indeed be limited, by the *Wld*^*S*^ protein following optic nerve crush and lens injury. It will be important to investigate in future studies the mechanisms that underlie these effects, and to identify combination treatments that both protect cell bodies and enhance RGC axon regeneration in *Wld*^*S*^ transgenic rats.

## Methods

### Surgical procedures

#### Animals

All animal experiments were conducted in accordance with the U.K. Home Office regulations for the care and use of laboratory animals, the U.K. Animals (Scientific Procedures) Act (1986) and the ARVO Statement for the Use of Animals in Ophthalmic and Vision Research.

Homozygous young adult, 2-3 month old, male *Wld*^*S*^ transgenic rats (n = 31) from line 79, in which the *Wld*^*S*^ cDNA transgene is driven by a β-actin promoter [20,25], or age-matched Sprague Dawley rats (n = 27; Charles River, Margate, UK) which are the background strain the *Wld*^*S*^ rats arose from [20], were used for this study. Animals were kept in a pathogen controlled environment on a 12 hour light–dark cycle in standard cages and allowed to feed and drink *ad libitum*.

For *in vivo* experiments rats were assigned to three groups: optic nerve crush (n = 8; Sprague Dawley; n = 9; *Wld*^*S*^), optic nerve crush + lens injury (n = 11; Sprague Dawley; n = 13; *Wld*^*S*^), untreated (control; n = 8 Sprague Dawley; n = 9; *Wld*^*S*^).

#### Optic nerve crush and lens injury

Rats were anesthetized by intraperitoneal injection of ketamine (75 mg/kg) and domitor (medetomidine hydrochloride; 0.5 mg/kg). Analgesics (Buprenorphine 0.03 mg/kg) were administered pre- and post-surgery. Unilateral optic nerve crush was performed as previously described [1], avoiding injury to the retinal artery in the dural sheath (confirmed by indirect ophthalmoscopy) by incising the dorsum of the latter longitudinally before crushing the optic nerve. The lens was injured at the time of the optic nerve crush by inserting the tip of a 25 gauge needle into the eye 2 mm anterior to the nerve head, perpendicular to the sclera to puncture the lens to a depth of about 2 mm. Lens injury was confirmed by direct visualization through the cornea.

### Immunohistochemistry on tissue sections

Animals were transcardially perfused 14 days post-injury under terminal anaesthesia with 0.1 M phosphate buffered saline (PBS) followed by 4% paraformaldehyde. Eyes and optic nerves were removed and post-fixed by immersion overnight in cold 4% paraformaldehyde. Tissue was then washed with PBS and transferred to 30% sucrose solution (overnight at 4 °C) for cryoprotection and embedded in OCT mounting medium (Raymond A. Lamb, Eastbourne, UK). Serial sections, 14 μm thick, were cut with a cryostat, thaw-mounted onto glass slides (Superfrost plus, VWR, Lutterworth, UK) and stored at -20 °C until further use.

Rehydrated eye sections were incubated in blocking solution (4% goat serum; 0.3% Triton (Sigma) in PBS) for 60 min at room temperature. Primary antibodies were applied overnight at 4 °C in blocking solution: mouse anti-Islet-1 homeobox (39.4D5 concentrate; 1:2000; hybridoma developed by T. Jessell and S. Brenner-Morton, concentrate from the Developmental Studies Hybridoma Bank, The University of Iowa, Iowa City, IA) and rabbit anti-GFAP (1:8000, DAKO, Ely, UK). Islet-1 is expressed by RGC, as well as by displaced amacrine cells in the ganglion cell layer [15,16]. GFAP is a marker for activated astrocytes/Müller glia [32]. The following day, slides were washed 3 x 10 min in PBS and secondary antibodies (Alexa Fluor 488 goat anti-rabbit IgG; 1/1000 and Alexa Fluor 555 goat anti-mouse IgG; 1/1000, Invitrogen, Paisley, UK) were applied for 2 hrs at room temperature in blocking solution. Following 3 x 10 min washes in PBS, slides were mounted in Fluorsave (Calbiochem/Merck Chemicals, Beeston, UK).

Immunohistochemistry was carried out to visualize growth-associated membrane phosphoprotein-43 (GAP-43) positive axons in optic nerve sections as previously described [2]. GAP-43 is normally not detected in the mature optic nerve, but it is strongly upregulated in regenerating RGC to fill the somata and axons [1,2,33]. As primary antibody the IgG fraction from an anti-GAP-43 antiserum prepared in sheep (1:2500; [34]) followed by an Alexa Fluor 488 secondary antibody against sheep IgG (1:1000, Invitrogen) were used.

### Quantification of RGC survival/axon regeneration and retinal glia activation

Quantification of GAP-43 positive RGC axon growth in the distal optic nerve stump was based on a previously described method [1]. Briefly, axon growth was quantified by counting the number of GAP-43 positive axons extending 250 μm from the crush site into the distal optic nerve in four longitudinal sections per optic nerve, using a standard epifluorescence microscope (model DM6000B; Leica, Wetzlar, Germany). The cross-sectional width of the nerve was measured at the point at which the counts were taken and was used to calculate the number of axons per millimetre of nerve width. The number of axons was then averaged over the four sections. ∑a_d_, the total number of axons extending distance *d* in a nerve having a radius of *r*, was estimated by summing over all sections having a thickness *t* (14 μm): ∑a_d_ = πr^2^ x [average axons/mm]/t.

To evaluate RGC survival, the number of Islet-1+ cells were counted in 4 sections throughout the eye and normalised to 100 μm retinal section length. Results are expressed as percentage difference versus control (control set as 100% baseline). For measuring activation of retinal glia, images of GFAP staining were taken along the retina in 4 sections throughout the eye, and the pixel intensity quantified using the Leica Application Suite (LAS AF.1.8.0) program. Results are expressed as percentage difference in GFAP activation versus control (control set as 100% baseline).

Results are expressed as mean ± SEM of 8-13 rats per condition for each of the experimental groups. The significance of intergroup differences was evaluated by an unpaired two-tailed *t* test (assuming equal variances) and considered significant at P < 0.05 (P < 0.05 = *; P < 0.01 = **; P < 0.001 = ***).

## Abbreviations

CNS: Central nervous system; GAP-43: Growth-associated membrane phosphoprotein-43; GFAP: Glial fibrillary acidic protein; Nmnat1: Nicotinamide mononucleotide adenylyl-transferase-1; PBS: Phosphate buffered saline; PNS: Peripheral nervous system; RGC: Retinal ganglion cell; SEM: Standard error of the mean; WldS: Slow Wallerian degeneration mutation.

## Competing interests

The authors declare that they have no competing interests.

## Author’s Contributions

BL designed the study and drafted the manuscript. She also carried out surgical procedures, tissue processing and parts of the immunohistochemistry and data collection, and performed data analysis. AT did the majority of immunohistochemistry and data collection. NDB bred the *Wld*^*S*^ transgenic rats. MM performed some data collection. KRM conceived of the study, participated in its design and helped to draft the manuscript. All authors read and approved the final manuscript
